# Landscape of clinical drug development of ADCs used for the pharmacotherapy of cancers: an overview of clinical trial registry data from 2002 to 2022

**DOI:** 10.1186/s12885-024-12652-5

**Published:** 2024-07-26

**Authors:** Wenjing Zhou, Zhiyuan Xu, Shu Liu, Xiaohuan Lou, Pengcheng Liu, Huali Xie, Shuiyan Zhang, Xi Liu, Baoshan Zhuo, Hongbing Huang

**Affiliations:** 1 Department of Clinical Trials Center, The University of Hong Kong-Shenzhen Hospital, Haiyuan 1st Road, Futian District, Shenzhen, 518000 P. R. China; 2 Department of Clinical Oncology Center, The University of Hong Kong-Shenzhen Hospital, Shenzhen, 518000 P. R. China; 3 State Key Laboratory of Oncology in South China, Guangdong Provincial Clinical Research Center for Cancer, Sun Yat-sen University Cancer Center, Guangzhou, 510060 P. R. China; 4 School of International Pharmaceutical Business, China Pharmaceutical University, Nanjing, 211198 P. R. China; 5 Department of Pharmacy, The University of Hong Kong-Shenzhen Hospital, Shenzhen, 518000 P. R. China; 6 Department of Chinese Medicine, The University of Hong Kong-Shenzhen Hospital, Shenzhen, 518000 P. R. China

**Keywords:** Antibody-drug conjugate, ADC, Clinical trials, Payload, Cancer

## Abstract

**Background:**

To provide reference for clinical development of ADCs in the industry, we analyzed the landscape and characteristics of clinical trials about antibody-drug conjugates (ADCs).

**Method:**

Clinical trials to study ADCs used for the pharmacotherapy of cancers initiated by the sponsor were searched in the Cite line Pharma Intelligence (Trialtrove database), and the landscape and characteristics of these clinical trials were analyzed from multiple perspectives, such as the number, phases, status, indications, and targets of the clinical trials.

**Result:**

As of December 31, 2022, a total of 431 clinical trials have been initiated to study ADCs used for the pharmacotherapy of cancers, and the number of the last 10 years was 5.5 times as large as the first 11 years. These clinical trials involved 47 indications, including breast cancer, lymphoma (lymphoma, non-Hodgkin’s and lymphoma, Hodgkin’s), unspecified solid tumor, bladder cancer and lung cancer (lung, non-small cell cancer and lung, small cell cancer). As for each of these five indications, 50 + clinical trials have been carried out, accounting for as high as 48.50% (454/936). ADCs involve 38 targets, which are relatively concentrated. Among them, ERBB2 (HER2) and TNFRSF8 (CD30) involve in 100 + registered clinical trials, and TNFRSF17 (BCMA), NECTIN4 and CD19 in 10 + trials. The clinical trials for these five targets account for 79.02% (354/448) of the total number. Up to 93.97% (405/431) of these clinical trials explored the correlation between biomarkers and efficacy. Up to 45.91% (292/636) of Lots (lines of treatment) applied in the clinical trials were the second line. Until December 31, 2022, 54.52% (235/431) of the clinical trials have been completed or terminated.

**Conclusion:**

ADCs are a hotspot of research and development in oncology clinical trials, but the indications, targets, phases, and Lot that have been registered are seemingly relatively concentrated at present. This study provides a comprehensive analysis which can assist researchers/developer quickly grasp relevant knowledge to assess a product and also providing new clues and ideas for future research.

**Supplementary Information:**

The online version contains supplementary material available at 10.1186/s12885-024-12652-5.

## Introduction

Generally, ADCs contain cytotoxic agent, linker and monoclonal antibody that is bound to the cytotoxic agent via a linker [1]. Combining the powerful killing effect of traditional chemotherapy drugs on cancer cells and precise targeting of antibody drugs [2, 3], ADCs can kill cancer cells in a targeted way, the systemic effects of the chemotherapy payload can be substantial but are overall reduced in frequency or severity or others compared to non-targeted delivery of chemotherapy, thereby achieving an anti-cancer therapeutic effect with high efficiency and low toxicity [4, 5].

As of December 31, 2022, 15 ADCs have been approved by different regulatory organizations worldwide. Indications for ADC drugs mainly included hematological tumors, breast cancer, gastric cancer, head and neck squamous cell carcinoma, etc., and the main targets include ERBB2, CD22 and TNFRSR 8 (Table 1).

**Table 1:** Marketed ADCs worldwide as of December 31, 2022

No.	Drugs	Company	Trade Names	Target antigens	Linkers	Payloads	Approved Countries	Approved Date	Approved Indications	First Approved Country
1	Gemtuzumab Ozogamicin	Pfizer	Mylotarg	CD33	hydrazone	N-acetyl-γ-Calicheamicin	FDA/EMA/PMDA	2000/05/17 2017/09/01	Leukemia, Acute Myelogenous	FDA
2	Brentuximab Vedotin	Seattle Genetics	Adcetris	TNFRSF8	mc-VC-PABC	MMAE	FDA/EMA/PMDA/NMPA	2011/08/19	Lymphoma, Hodgkin’s	FDA
3	Trastuzumab Emtansine	Roche	Kadcyla	ERBB2	SMCC	DM1	FDA/EMA/PMDA/NMPA	2013/02/22	Breast cancer	FDA
4	Inotuzumab Ozogamicin	Pfizer	Besponsa	CD22	hydrazone	N-acetyl-γ-Calicheamicin	FDA/EMA/PMDA/NMPA	2017/06/28	Leukemia, Acute Lymphocytic	EMA
5	Moxetumomab Pasudotox	AstraZeneca	Lumoxiti	CD22	mc-VC-PABC	PE38	FDA/EMA	2018/09/13	hairy cell leukemia, HCL	FDA
6	Polatuzumab Vedotin	Roche	Polivy	CD79B	mc-VC-PABC	MMAE	FDA/EMA	2019/6/10	Lymphoma, Non-Hodgkin’s	FDA
7	Enfortumab Vedotin	Seagen	Padcev	NECTIN4	mc-VC-PABC	MMAE	FDA	2019/12/18	Urothelial carcinoma	FDA
8	Trastuzumab Deruxtecan	Daiichi Sankyo	Enhertu	ERBB2	tetrapeptide	Dxd	FDA/EMA/PMDA	2019/12/20	Breast cancer	FDA
9	Sacituzumab Govitecan	Immunomedics	Trodelvy	TACSTD2	CL2A	SN-38	FDA/NMPA	2020/04/22	Breast cancer	FDA
10	Belantamab Mafodotin	GSK	Blenrep	TNFRSF17	mc	MMAF	FDA/EMA	2020/08/05	Multiple myeloma	FDA
11	Cetuximab Sarotalocan	Rakuten Medical	Akalux	EGFR	NA	IRDye700DX	PMDA	2020/09/25	Head/Neck	PMDA
12	Loncastuximab Tesirine	ADC Therapeutics	Zynlonta	CD19	dipeptide	PBD dimer(SG3199)	FDA	2021/04/23	Lymphoma, Non-Hodgkin’s	FDA
13	Disitamab vedotin	RemeGen	Aidixi	ERBB2	mc-VC-PABC	MMAE	NMPA	2021/06/08	Gastric	NMPA
14	Tisotumab vedotin	Seagen	Tivdak	F3	mc-VC-PABC	MMAE	FDA	2021/9/20	Cervical	FDA
15	mirvetuximab	ImmunoGen, Inc	Elahere	FOLR1	/	DM4	FDA	2022/11/14	Ovarian fallopian tube, or primary peritoneal cancer	FDA

Note: referring to FDA websites, company websites, press releases, conference reports

In 2011, a breakthrough in ADCs research was achieved when Adcetris (brentuximab vedotin) was approved by the FDA for the treatment of Hodgkin’s lymphoma (HL) and anaplastic large cell lymphoma (ALCL) [6, 7]. In 2013, Kadcyla (trastuzumab emtansine) was approved by the FDA for the treatment of ERBB2-positive metastatic breast cancer [8], making it the first ADCs for solid tumors. Although there are reviews on the current state of clinical development of clinical trials of ADCs, chemical structures of ADCs, and marketed ADC drugs [9–13], there is still a lack of articles that comprehensively describe the global development landscape of ADCs used for the pharmacotherapy of cancer. In this paper, the worldwide development landscape of ADCs clinical trials is summarized from multiple perspectives, including the number, phase, status, indication and targets of clinical trials to study ADCs used for the pharmacotherapy of cancer, which can help researchers understand the current research hot spots in this field, explore potential research directions, and identify future development trends.

## Materials and methods

### Source of data

The research group chose Citeline Pharma Intelligence, which gathers information of clinical trials worldwide and is a timely updated database, as data source [14–16]. Then research group used cross-checking data with multiple published databases, including clinical Trials.gov, European Clinical Trials Register, China Chinese Clinical Trial Registry, etc. Company websites, press releases, and academic publications were also consulted to confirm drug information. Search conditions were clinical trials with tested drug that includes “Antibody-Drug Conjugate” or “ADC”, “Sponsor Type is Industry” and “Therapeutic Area is Oncology”. A total of 548 clinical trials as of June 30, 2023 were searched, with relevant information obtained. Of these, 40 clinical trials without definite start date and 1 with “Other” clinical phase were excluded, thus yielding 507 clinical trials. After checking by two investigators, 44 clinical trials with tested drugs that do not include ADCs were excluded, thus leaving 463 clinical trials. As the full-year information of 2023 was not available as of the data collection date, 32 clinical trials carried out in 2023 were excluded. Finally, a total of 431 ADCs clinical trials were analyzed in this paper.

### Methods

Based on the retrieved data, frequency distribution analysis and graph analysis were carried out according to the trial phase, trial status, indication distribution, target distribution, chemotherapy line of investigational drug, country of sponsor, and whether biomarker studies were carried out. The study spans 21 years from 2002 to 2022, and research group chose to compare before and after the center of the time period. There were only two ADCs on the market in the 11-year period from 2002 to 2012, whereas in the 10-year period from 2013 to 2022, there are already 13 ADCs on the market. So the period from 2002 to 2022 is divided into two periods, which is 2002–2012 and 2013–2022. The rate variance was analyzed with Chi-square test by using SPSS 29. For the indication-target relationship, an Upset map was used to show the distributions and overlap of targets in each kind of indications (top 10) and to show the intersection of multiple collections to highlight the overlap of targets between different indications. The “set size” is the targets number and the “indication size” is the top 10 malignancy type in this upset map.

## Result

### Number and phase of ADC clinical trials

**Fig. 1:**
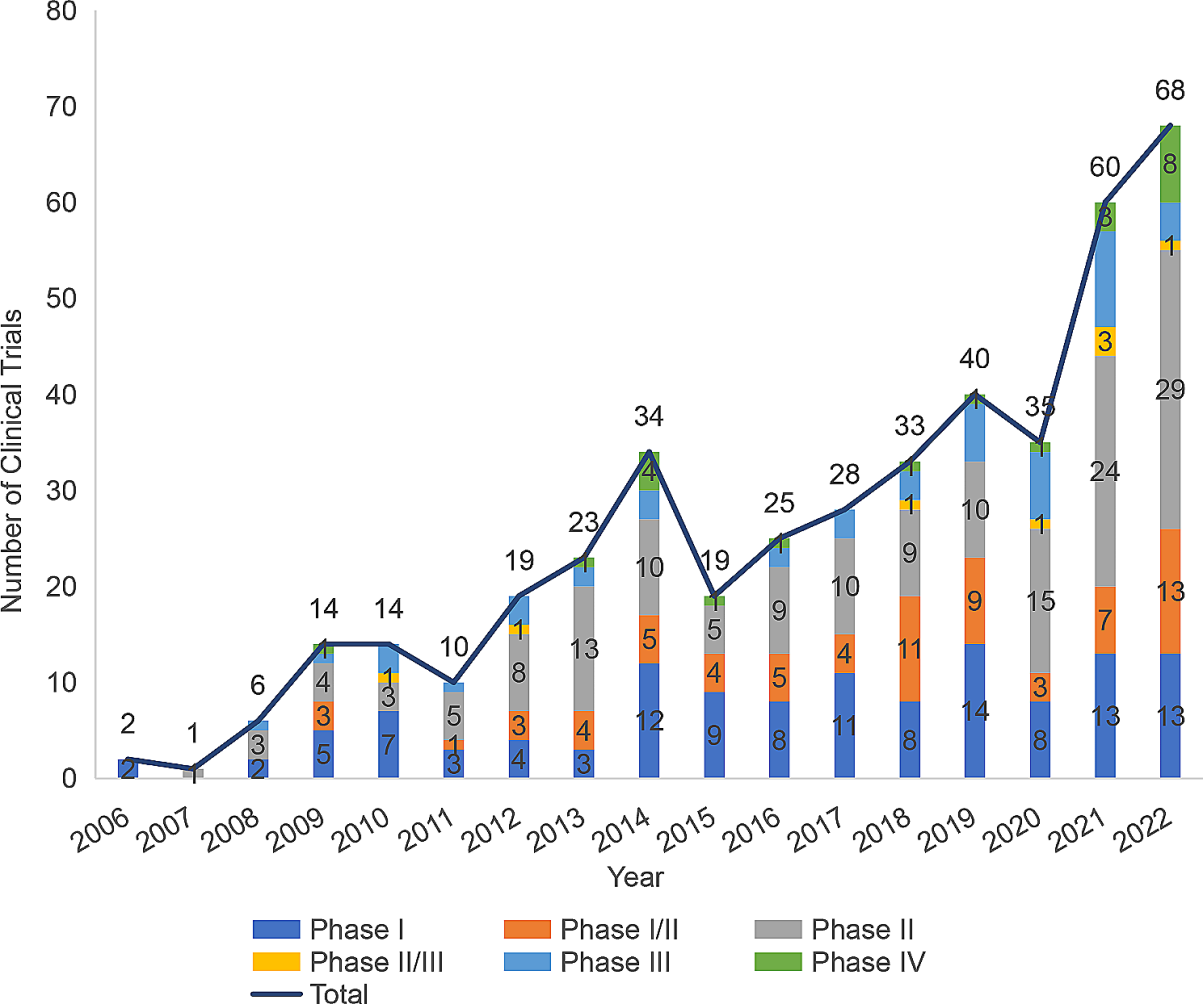
As of December 31, 2022, the annual distribution of clinical trials of ADCs by Phase

As of December 31, 2022, a total of 431 ADCs clinical trials initiated by global enterprises have been registered, showing an ever-growing trend, and peaking (68) in 2022. These registered clinical trials were mainly in Phases I and II, as shown in Fig. 1.

### Indications and targets of ADC clinical trials

#### Indications of ADCs clinical trials

**Fig. 2:**
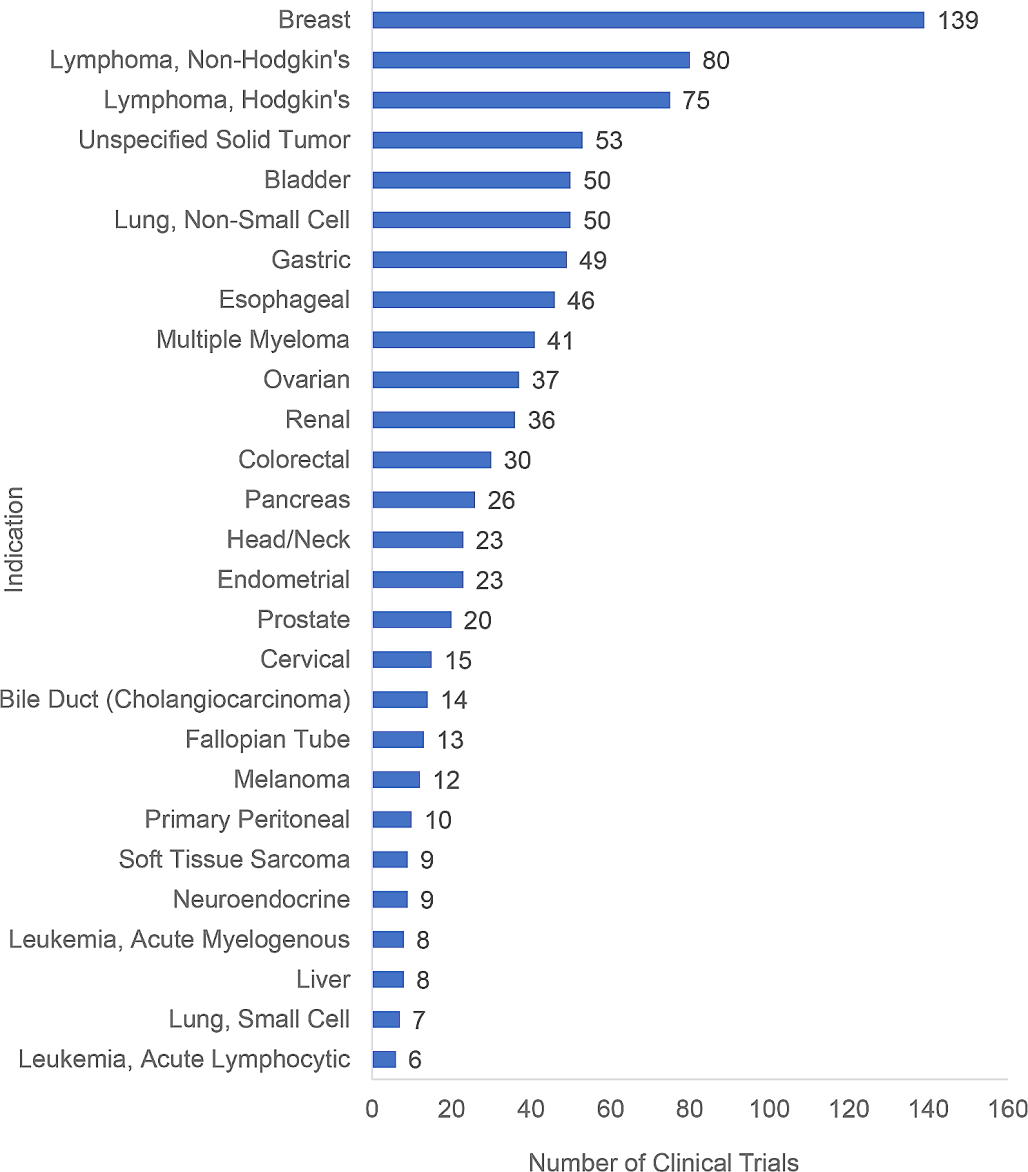
As of December 31, 2022, ADCs clinical trial indications distribution Note: If patients with multiple indications are enrolled, one clinical trial will be recorded for each of the corresponding indications. Due to the limited space, only tumor types involved in 5 or more clinical trials are shown

Frequency distribution analysis and graph analysis for ADCs clinical trials were carried out by indications. Indications mainly included breast cancer 14.85% (139/936), lymphoma, non-Hodgkin’s 8.55% (80/936), lymphoma, Hodgkin’s 8.01% (75/936), unspecified solid tumor 5.67% (53/936), bladder cancer 5.34% (50/936) and non-small cell lung cancer 5.34% (50/936). As some clinical trials enrolled patients with multiple indications, the total number of clinical trials by indications was 936, which was greater than the number of registered clinical trials 431. As shown in Fig. 2.

#### Target distribution of ADCs

**Fig. 3:**
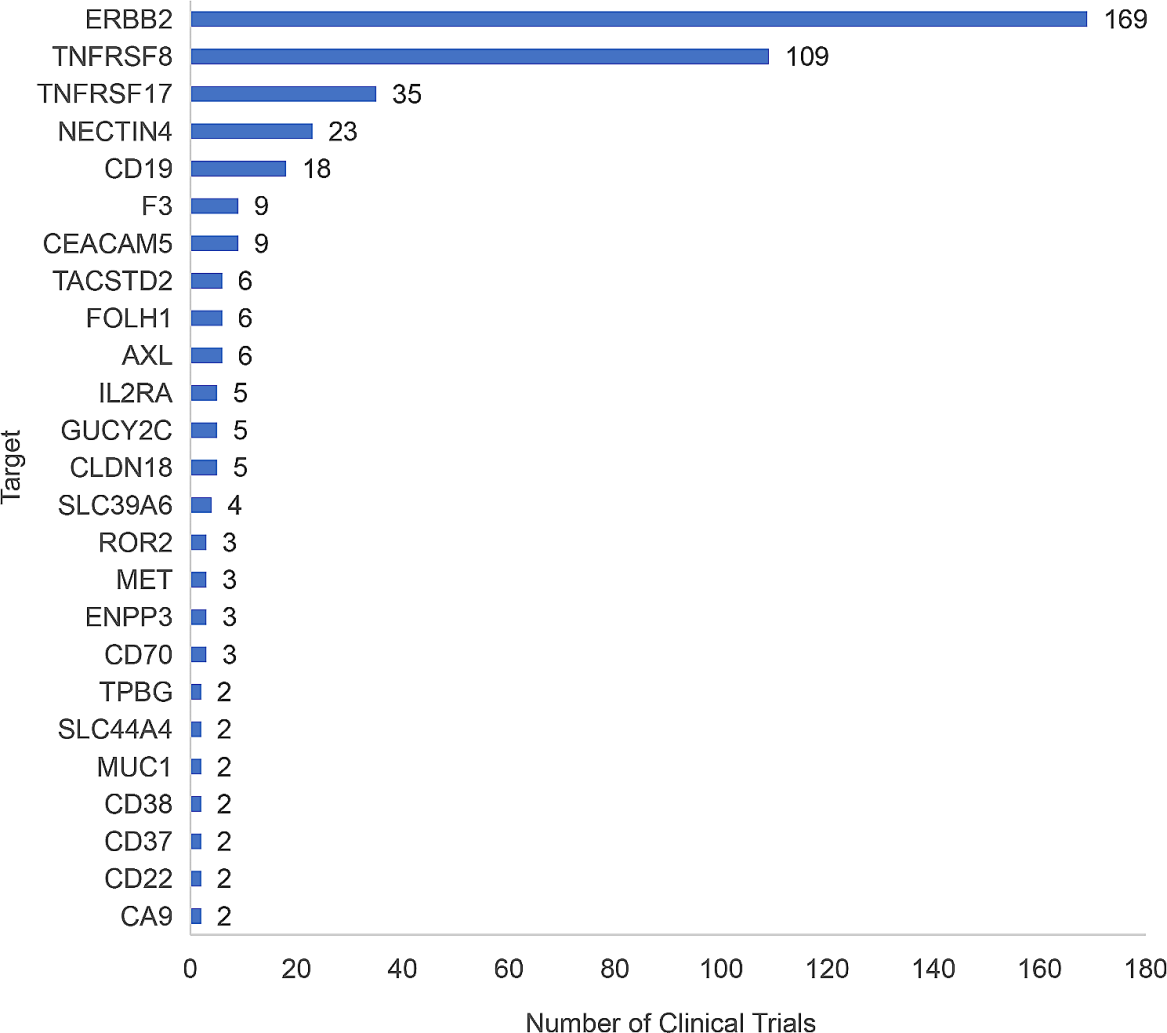
As of December 31, 2022, ADCs clinical trial target distribution Note: If an investigational drug has multiple targets, one clinical trial will be recorded for each of the targets. Due to the limited space, only targets involved in at least 2 clinical trials are shown

Frequency distribution analysis and graph analysis for ADCs clinical trials were carried out by targets. Top three targets were ERBB2 37.72% (169/448), TNFRSF8 24.33% (109/448), and TNFRSF17 7.81% (35/448). As investigational drugs used in some clinical trials had multiple targets, the total number of clinical trials by targets was 448 according to the statistics, greater than the number of registered clinical trials (431). As shown in Fig. 3.

#### Indication-target relationship in ADCs clinical trials

**Fig. 4:**
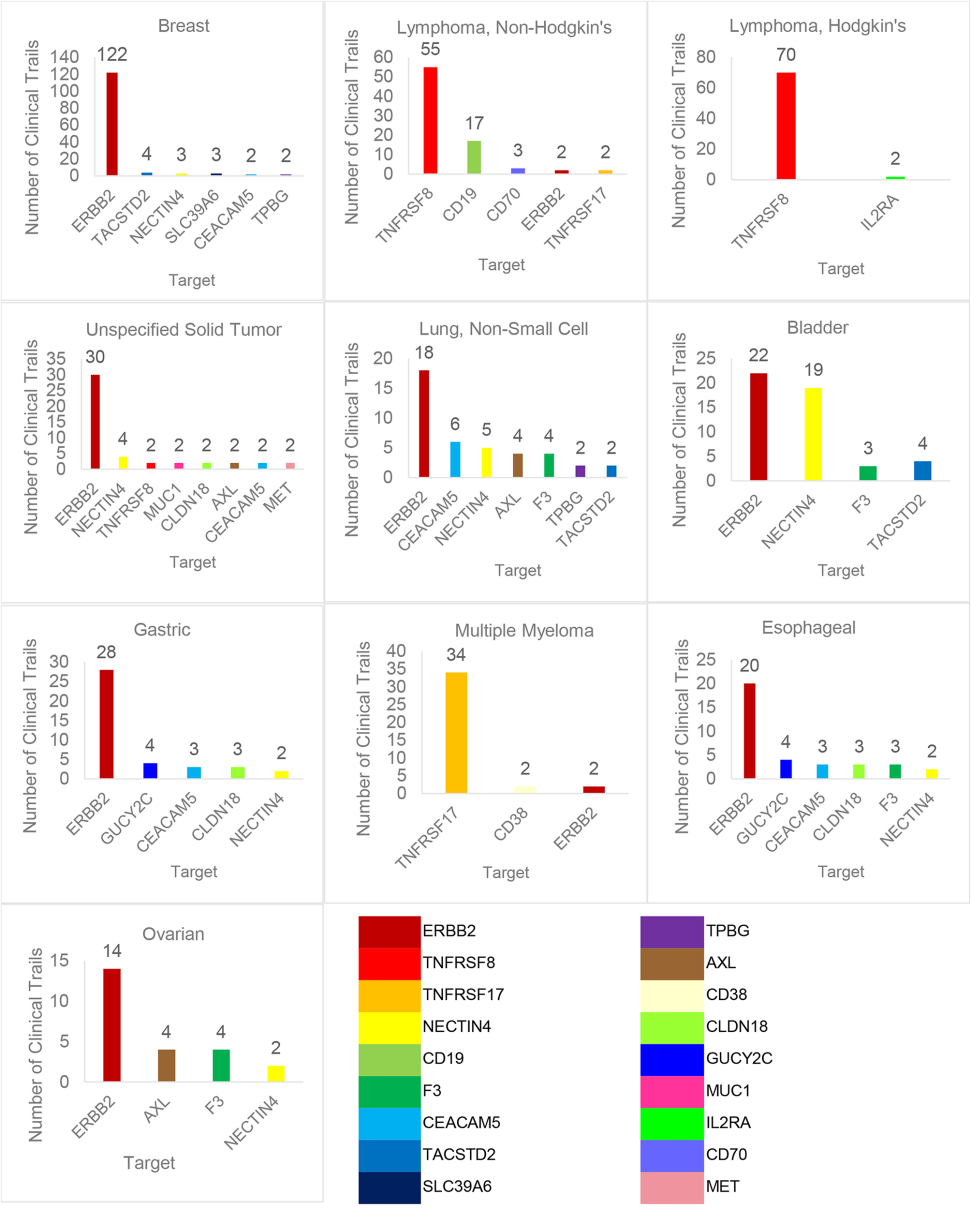
Target distribution of ADCs corresponding to top 10 indications Note: Due to the limited space, only the top 10 tumor types in quantity and targets when at least 2 ADCs are used are shown

As shown in Fig. 4 and Table S1(Table Supplymentary 1), the top 5 indications currently treated with ADCs in clinical studies are breast cancer, non-Hodgkin lymphoma, Hodgkin lymphoma, unspecified solid tumor and bladder cancer.

**Fig. 5:**
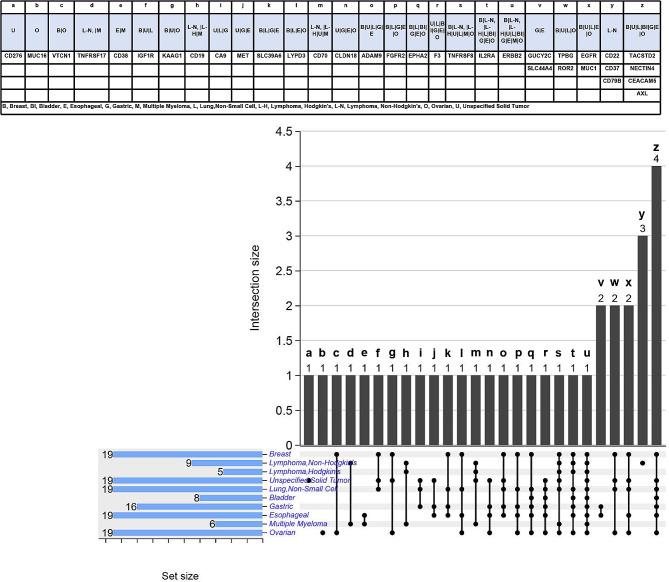
Upset map of target crossover of ADCs in top 10 malignancy type Note: UpSet plot for the distributions of targets in each kind of malignancy type (top 10). In the matrix of dots in the bottom‑right hand corner each row represents an malignancy type and each column represents a subset of targets. Above the matrix of dots, a vertical bar chart represents the number of targets in each subset. To the left of the matrix of dots, a horizontal bar chart represents the number of targets including each specific malignancy type. The solid vertical lines connecting dots within a column indicate which malignancy type are included in each subset of targets. If a subset of targets consists of a single malignancy type there is no vertical line. For example, for the last solid vertical lines connecting dots, the number of the corresponding vertical bar chart was 4, represented this subset contains four targets, and these four targets appeared simultaneously in seven malignancy type. The above table shows in detail the targets represented by each column and the overlap of targets in each kind of indications in the same order with this figure. The UpSet plot was mapped through online sites https://www.chiplot.online/

As shown in Fig. 5, different malignancy type share the same target. There are 9 targets that involve 5 + malignancy type concurrently, 4 targets with the most repetitions co-exist in 7 tumor types, and ERBB2 is involved in 10 malignancy type. Only individual targets of non-Hodgkin lymphoma, non-specific solid tumor and ovarian cancer are exclusive.

### Antibodies and potent cytotoxicity payloads of ADCs

**Fig. 6:**
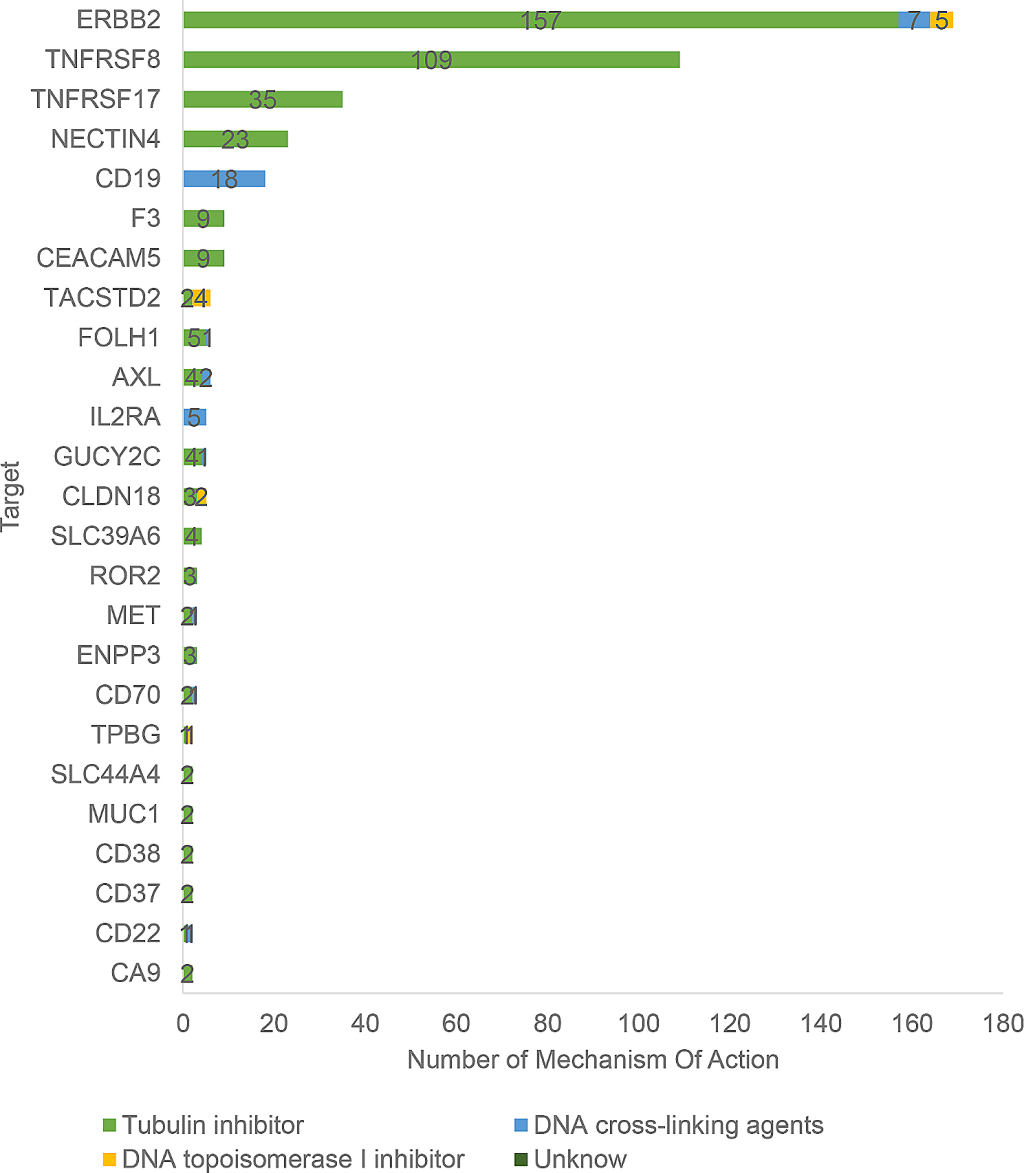
Molecular mechanism distribution of ADCs targets and cytotoxicity components

Cytotoxicity components connected to ADCs are divided into microtubule inhibitors, DNA cross-linking agents, and DNA topoisomerase inhibitors by their molecular mechanisms. As shown in Fig. 6, a vast majority of ADCs are bridged with microtubule inhibitors (88.17%, 395/448), while CD19 and IL2RA target drugs are mainly bridged with DNA cross-linking agents.

### Registered LoTs of ADCs clinical trials

**Fig. 7:**
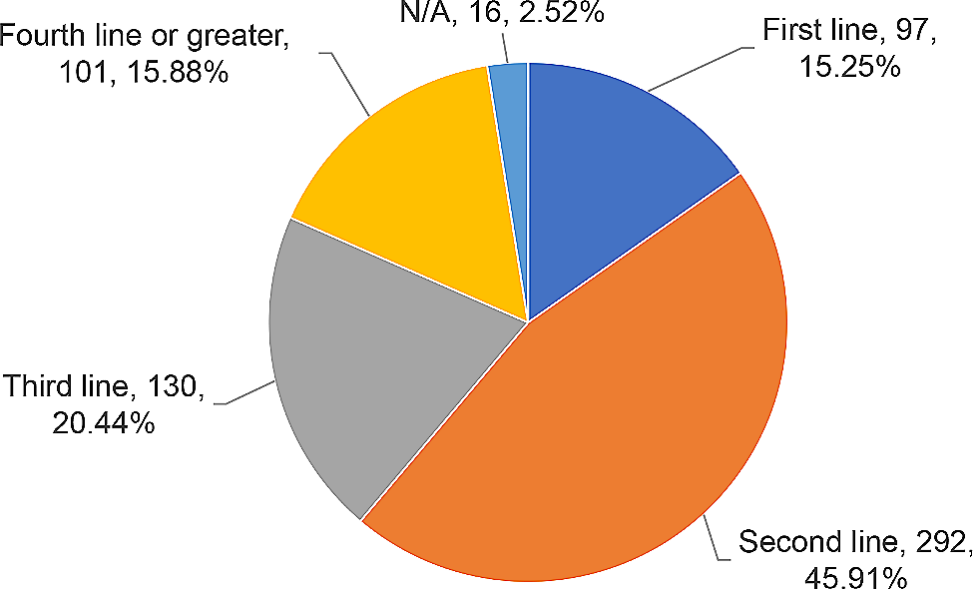
Global ADCs clinical trial lines of treatment distribution as of December 31, 2022 Note: If an investigational drug corresponds to multiple chemotherapy lines, one clinical trial will be recorded for each of the corresponding chemotherapy lines. N/A indicated that the data was not registered by the sponsor and was shown as missing in the database

Frequency distribution analysis and graph analysis were carried out by chemotherapy line of investigational drugs used in ADCs clinical trials. Among these investigational drugs, 45.91% (292/636) were for the second-line therapy, 20.44% (130/636) for the third line, 15.88% (101/636) for the fourth line or greater, and 15.25% (97/636) for first line. Given the fact that investigational drug used in a clinical trial might correspond to multiple chemotherapy lines, the total number 636 of chemotherapy lines was greater than the number of registered clinical trials 431. As shown in Fig. 7.

### Biomarkers for clinical trials of ADCs

**Fig. 8:**
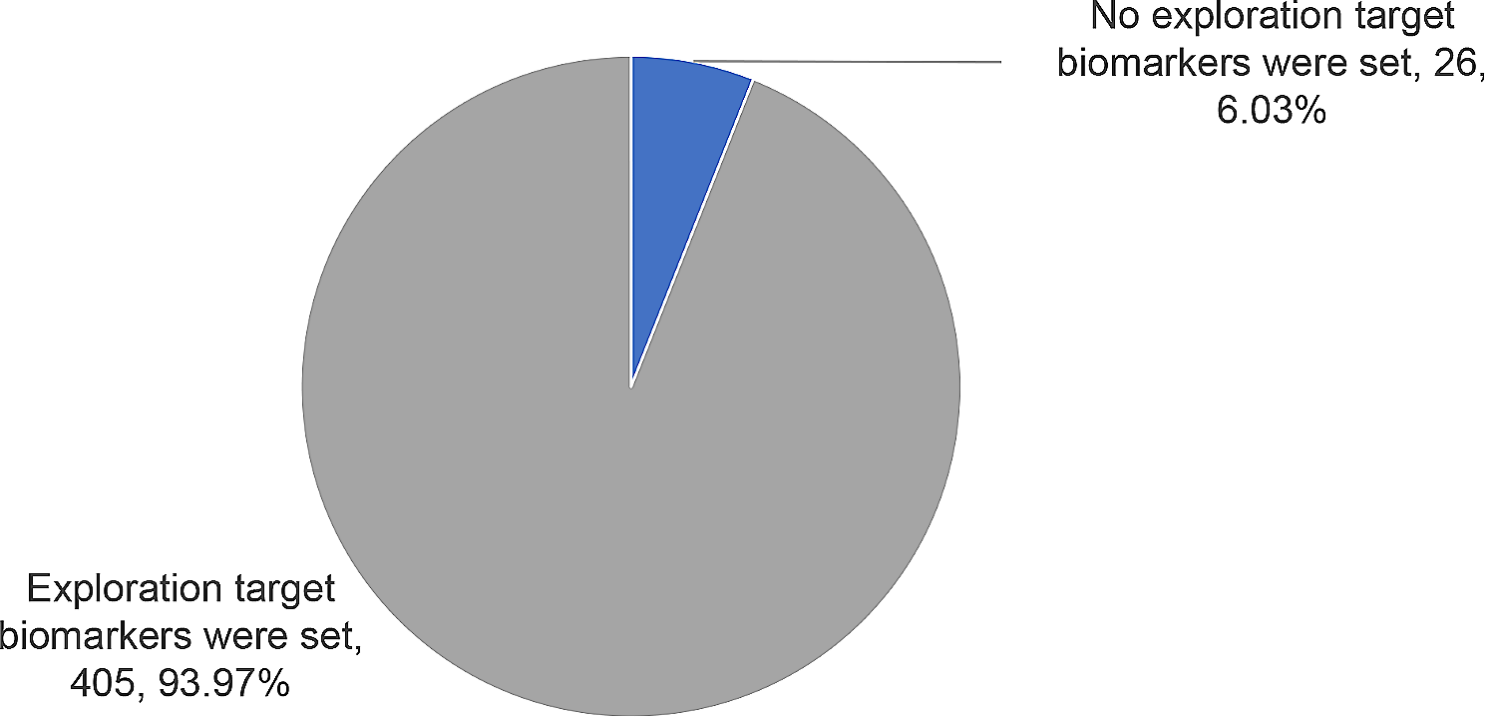
As of December 31, 2022, exploration of biomarkers in ADCs clinical trials

It can be found that 93.97% (405/431) of the ADCs clinical trials initiated by global enterprises have carried out investigations on biomarkers and efficacy. As shown in Fig. 8.

### Statistical analysis by status of ADCs clinical trials

**Fig. 9:**
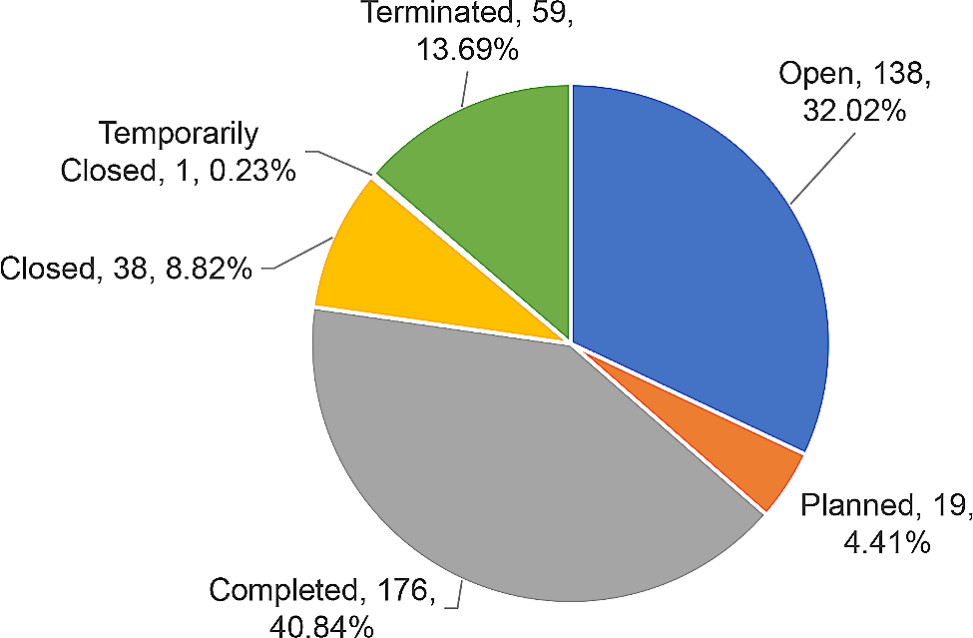
As of December 31, 2022, ADCs clinical trial status

Among ADCs clinical trials initiated by global enterprises, 32.02% (138/431) were open, 40.84% (176/431) have been completed, 4.41% (19/431) were in the planning stage, and 13.69% (59/431) have been terminated. As shown in Fig. 9.

### Changes in country of sponsor, phase registered and LoT of ADCs clinical trials during different time periods

The country of sponsor, phase registered and LoT of the ADCs clinical trials were compared in two time periods: 2002–2012 (*n* = 66) and 2013–2022 (*n* = 365). Chi-square test was used for variance analysis, as shown in Table 2.

**Table 2:** Trend changes in characteristics of ADCs trials from 2002–2012 to 2013–2022

Characteristic	No./total no. (%)		*P* Value
	2002–2012	2013–2022	
Sponsor country			
United States	32/66(48.5)	110/365(30.1)	<0.01
Switzerland	25/66(37.9)	49/365(13.4)	<0.01
China	0/66(0.0)	63/365(17.3)	<0.01
Japan	5/66(7.6)	48/365(13.2)	0.20
United Kingdom	2/66(3.0)	37/365(10.1)	0.06
Phase			
1	23/66(34.8)	99/365(27.1)	0.20
1/2	7/66(10.6)	65/365(17.8)	0.15
2	24/66(36.4)	134/365(36.7)	0.96
2/3	2/66(3.0)	6/365(1.6)	0.35
3	9/66(13.6)	40/365(11.0)	0.53
4	1/66(1.5)	21/365(5.8)	0.22
Patient Segment			
First line	20/115(17.4)	77/521(14.8)	0.48
Second line	47/115(40.9)	245/521(47.0)	0.23
Third line	23/115(20.0)	107/521(20.5)	0.90
Fourth line or greater	23/115(20.0)	78/521(15.0)	0.18

Note: 1. Sixteen clinical trials lack Patient Segment information; 2. Only Top 5 sponsor countries are included into statistics

From 2002–2012 to 2013–2022, the number of ADCs clinical trials continued to increase in countries. Among them, China, Japan and the UK have achieved great breakthroughs, and their proportion has increased sharply over the past one decade. These clinical trials are mainly in phases I ~ II. The LoTs applied are mainly the second line, accounting for 45.91% (292/636) of the total ADCs clinical trials.

## Discussion

From 2002–2012 to 2013–2022, the number of ADCs clinical trials increased from 66 to 365, the last 10 years were 5.5 times as large as the first 11 years, indicating that ADCs have gradually been development hotspot of cancer pharmacotherapy [17, 18]. In addition, the US is the country initiating the most (32.95%) ADCs clinical trials, followed by Switzerland (17.17%) and China (14.62%).

Our analysis shows that the indications and targets of ADCs clinical trials are relatively concentrated. Indications of the 15 ADCs that have been approved by different regulatory organizations worldwide including lymphoma, breast cancer, urinary system tumors, head and neck tumors, gastric cancer, and gynecological tumors and so on, as shown in Table 1. Among the 431 registered clinical trials, 49.52% applied the same indication. Nonetheless, other indications were also explored over the past few years, such as esophageal cancer, intestinal cancer, pancreatic cancer, liver cancer/cholangiocarcinoma, melanoma, soft tissue sarcoma and neuroendocrine tumors. Currently, pancreatic cancer and soft tissue sarcoma still have poor prognosis with limited approved targeted drugs, and the success in clinical trials may lead to a major breakthrough in ADCs and even targeted therapy for these tumor types [19, 20]. Addressable indications and target antigen are pivotal challenges for the long-term growth in the application of ADCs, this study provides a comprehensive analysis which can assist researchers/developer quickly grasp relevant knowledge and to explore the advantages or risks for a product.

Our study data also showed that 34.80% (150/431) of ADCs clinical trials explored multiple indications simultaneously. Besides, 53 ADCs clinical trials involved the unspecified solid tumors. After manual screening by our research group, 29 of them were designed as basket trials. This suggests that basket design may become increasingly popular in the development of trials for ADCs. It classifies indications by targets, rather than by traditional classification of indications by primary tumor sites and pathological patterns. At the same time, attention shall be paid to avoiding the common drawbacks of basket-type clinical trials, such as sample size and the design of control cohort [21, 22].

Taking ADCs targeting ERBB2 as an example, in theory, they should be effective against ERBB2 over-expressed/mutated cancers, including breast cancer, gastric cancer, urothelial carcinoma, colorectal cancer, and non-small cell lung cancer [23–27]. However, existing literature reveals that different ADCs targeting the ERBB2 may exhibit not exactly same pharmacokinetics (PK), pharmacodynamics (PD), efficacy and safety characteristics [28, 29]. For example, Kadcyla (Trastuzumab emtansine/T-DM1)is effective in the treatment of ERBB2-positive breast cancer, but it has not delivered remarkable clinical benefit in ERBB2-positive gastric cancer [30]. In contrast, a phase III open-label randomized controlled trial showed that Enhertu (Trastuzumab deruxtecan/T-DXd) was effective in patients with ERBB2 under/over-expressed breast cancer and gastric cancer [31, 32]. T-DM1’s payload consists of the cytotoxic microtubule inhibitor(DM1), with a drug-to-antibody ratio (DAR) of 3.5:1. T-DXd’s payload in this case is an Exatecan derivative which acts through topoisomerase I inhibition, with a DAR of 8:1 [33]. This indicates that their payload was substantially different. ADCs featuring cleavable linkers and membrane-permeable payloads have been shown to exhibit additional activity via the bystander effect, providing indiscriminate cytotoxicity after penetrating tumors and overcoming tumor heterogeneity [33, 34]. However, the clinical efficacy of ADCs is also influenced by the antibody, linker and payload, each influenced by complex interactions between the ADC and various components within both the tumour itself as well as microenvironment (TME) [35].

This suggests that a drug that acts on the same targetmay present subjects with different benefits. Therefore, it is recommended that the efficacy, safety and the rationality of dosing regimen of ADCs for different indications should be adequately investigated in early clinical trials, to provide sufficient basis for determining the target indication.

The study also suggests that the targets of ADCs clinical trials are relatively concentrated. The 15 ADC drugs that have been approved by different regulatory organizations worldwide involve 12 targets referred to Table 1. Of the 431 clinical trials analyzed in this study, up to 373 trials, or 83.26% (373/448), have overlapped drug targets. For example, there are 149 breast cancer ADCs have been studied in clinical trialsinvolving a total of 19 targets. Among them, 122 ADCs target ERBB2 as shown in Table Supplymentary 1. Currently, three ADCs targeting ERBB2 had successfully hit the market. Having said that, efforts have also been made to explore emerging targets. For example, there were currently 9 trials involving CEACAM5, a tumor marker over-expressed on the surface of multiple solid tumors, such as lung cancer, colorectal cancer and gastric cancer. Drugs developed for this disease include monoclonal antibody, bispecific antibody, chimeric antigen receptor T cell (CAR-T) and tri-specific antibody [36]. The 2023 European Lung Cancer Congress (ELCC 2023) published that the monotherapy of CEACAM5-targeting ADC SAR408701 (Tusamitamab Ravtansine) had showed antitumor activity and safety in patients with CEACAM5-positive non-squamous non-small cell lung cancer who have received multiple treatment regimens. Therefore, it was expected to be the first drug to hit the market among the like, opening up a new target drug market [37].

In terms of clinical trial phasing and precautions, 81.67% (352/431) of global ADCs clinical trials were still in the early clinical research phase (Phase I, Phase I/II and Phase II) and the LoTs applied were mainly the second line referred to Table 2. This suggests that the optimal dosing strategies should be taken into consideration comprehensively in the design of most ADCs clinical trials. Assessing the exposure of ADC and its components in relation to safety and/or efficacy can support the selection of dosing strategies through pivotal research. For ADCs that cause principal toxicities due to excessive total antibody Peak Concentration (Cmax) in serum, an effective low-dose fractionated dosing strategy can be used [38–43]. For example, Mylotarg (Gemtuzumab Ozogamicin), which was initially approved for the treatment of CD33-positive acute myeloid leukemia (AML) in 2001 [44], withdrew from the market in 2010 due to severe toxicity [39, 45]. After the introduction of fractionated dosing, toxic reactions decreased, reducing Cmax in serum, thus enhancing safety and tolerability [46]. Therefore, it is suggested that the PK and PD of ADCs, ingredients and pharmacologically active metabolites are fully understood in the early stage of research, with the correlation between them and safety/efficacy being explored as far as possible, to adjust administration regimen in a timely, reasonable way. This study also provided an overview about cytotoxic components, chemotherapy lines, biomarkers, and so on, to assist researchers to explore the evolution and development trends of research hotspots in this field and also providing new clues and ideas for future research.

## Conclusion

Data of this study mainly come from the Citeline Pharma Intelligence. This database incorporates clinical trial registration databases from more than 70 countries (and regions), such as the US ClinicalTrials.gov [47], European Clinical Trials Register [48], Japan Pharmaceutical Information Center [49], China Chinese Clinical Trial Registry [50], as well as other sources of clinical information (e.g., sponsor/corporate registries, partner groups, and major medical centers). All trial information was included in the corresponding drug and clinical trial database in the form of code, providing complete information for traceability to make verification transparent. Limited by the search method, it may not incorporate all clinical trials of ADCs worldwide, and clinical trial results were missing from the database, so we did not have further analysis of ADCs and clinical efficacy.

In summary, ADCs is hotspot in precision treatment of cancer in addition to immunotherapy and Tyrosine kinase inhibitors (TKI) targeted therapy [33].

However, we summarized comprehensively the worldwide development landscape of ADC clinical trials from multiple perspectives, including the number, phase, status, indication and targets of such trials, with suggestions proposed, providing scientific reference for ADCs development institutes, enterprises and medical practitioners. From the perspective of industry development, to make ADCs hit the market successfully, it is imperative to optimize the drug design according to the characteristics of ADCs and tumors based on patients’ clinical demands.

### Electronic supplementary material

Below is the link to the electronic supplementary material.

Supplementary Material 1

## Data Availability

The datasets generated during and/or analysed during the current study are available in the Citeline Pharma Intelligence (Trialtrove database) repository, https://www.citeline.com.

## Abbreviations

ADCs: Antibody-Drug Conjugates
FDA: Food and Drug Administration
HL: Hodgkin Lymphoma
ALCL: Anaplastic Large Cell Lymphoma
EMA: European Medicines Agency
PMDA: Pharmaceuticals and Medical Devices Agency
NMPA: National Medical Products Administration
ELCC: The European Lung Cancer Congress
PK: Pharmacokinetic
PD: Pharmacodynamic
LoTs: Lines of Treatment
