# Supplementary material for: Landscape of clinical drug development of ADCs used for the pharmacotherapy of cancers: an overview of clinical trial registry data from 2002 to 2022
Source: BMC Cancer. 2024 Jul 26;24:898. doi: 10.1186/s12885-024-12652-5 (PMC11282866; doi:10.1186/s12885-024-12652-5)
Supplement: Supplementary file 1 — Supplementary Material 1 [file 12885_2024_12652_MOESM1_ESM.docx]

Table S1 Target distribution of ADCs corresponding to top 10 diseases

Note: Due to the limited space, only the top 10 tumor types in quantity are shown.

| **Disease** | **Target** | **Drug Number** | |
| --- | --- | --- | --- |
| **Breast** |  | | **149** |
|  | ERBB2 | | 122 |
|  | TACSTD2 | | 4 |
|  | NECTIN4 | | 3 |
|  | SLC39A6 | | 3 |
|  | CEACAM5 | | 2 |
|  | TPBG | | 2 |
|  | VTCN1 | | 1 |
|  | ADAM9 | | 1 |
|  | AXL | | 1 |
|  | EPHA2 | | 1 |
|  | EGFR | | 1 |
|  | MUC1 | | 1 |
|  | FGFR2 | | 1 |
|  | IGF1R | | 1 |
|  | IL2RA | | 1 |
|  | KAAG1 | | 1 |
|  | LYPD3 | | 1 |
|  | ROR2 | | 1 |
|  | TNFRSF8 | | 1 |
| **Lymphoma, Non-Hodgkin's** |  | | **83** |
|  | TNFRSF8 | | 55 |
|  | CD19 | | 17 |
|  | CD70 | | 3 |
|  | ERBB2 | | 2 |
|  | TNFRSF17 | | 2 |
|  | CD22 | | 1 |
|  | CD37 | | 1 |
|  | CD79B | | 1 |
|  | IL2RA | | 1 |
| **Lymphoma, Hodgkin's** |  | | **75** |
|  | TNFRSF8 | | 70 |
|  | IL2RA | | 2 |
|  | CD19 | | 1 |
|  | ERBB2 | | 1 |
|  | CD70 | | 1 |
| **Unspecified Solid Tumor** |  | | **57** |
|  | ERBB2 | | 30 |
|  | NECTIN4 | | 4 |
|  | TNFRSF8 | | 2 |
|  | MUC1 | | 2 |
|  | CLDN18 | | 2 |
|  | AXL | | 2 |
|  | CEACAM5 | | 2 |
|  | MET | | 2 |
|  | ROR2 | | 1 |
|  | CD70 | | 1 |
|  | ADAM9 | | 1 |
|  | CA9 | | 1 |
|  | F3 | | 1 |
|  | EGFR | | 1 |
|  | CD276 | | 1 |
|  | IGF1R | | 1 |
|  | KAAG1 | | 1 |
|  | TPBG | | 1 |
|  | TACSTD2 | | 1 |
| **Lung, Non-Small Cell** |  | | **53** |
|  | ERBB2 | | 18 |
|  | CEACAM5 | | 6 |
|  | NECTIN4 | | 5 |
|  | AXL | | 4 |
|  | F3 | | 4 |
|  | TPBG | | 2 |
|  | TACSTD2 | | 2 |
|  | ADAM9 | | 1 |
|  | CA9 | | 1 |
|  | EPHA2 | | 1 |
|  | EGFR | | 1 |
|  | FGFR2 | | 1 |
|  | IGF1R | | 1 |
|  | IL2RA | | 1 |
|  | LYPD3 | | 1 |
|  | MUC1 | | 1 |
|  | ROR2 | | 1 |
|  | SLC39A6 | | 1 |
|  | TNFRSF8 | | 1 |
| **Bladder** |  | | **52** |
|  | ERBB2 | | 22 |
|  | NECTIN4 | | 19 |
|  | F3 | | 3 |
|  | TACSTD2 | | 4 |
|  | AXL | | 1 |
|  | CEACAM5 | | 1 |
|  | EPHA2 | | 1 |
|  | IL2RA | | 1 |
| **Gastric** |  | | **51** |
|  | ERBB2 | | 28 |
|  | GUCY2C | | 4 |
|  | CEACAM5 | | 3 |
|  | CLDN18 | | 3 |
|  | NECTIN4 | | 2 |
|  | ADAM9 | | 1 |
|  | AXL | | 1 |
|  | CA9 | | 1 |
|  | F3 | | 1 |
|  | EPHA2 | | 1 |
|  | FGFR2 | | 1 |
|  | IL2RA | | 1 |
|  | MET | | 1 |
|  | SLC39A6 | | 1 |
|  | SLC44A4 | | 1 |
|  | TACSTD2 | | 1 |
| **Esophageal** |  | | **48** |
|  | ERBB2 | | 20 |
|  | GUCY2C | | 4 |
|  | CEACAM5 | | 3 |
|  | CLDN18 | | 3 |
|  | F3 | | 3 |
|  | NECTIN4 | | 2 |
|  | ADAM9 | | 1 |
|  | AXL | | 1 |
|  | CD38 | | 1 |
|  | EPHA2 | | 1 |
|  | EGFR | | 1 |
|  | FGFR2 | | 1 |
|  | IL2RA | | 1 |
|  | LYPD3 | | 1 |
|  | MET | | 1 |
|  | MUC1 | | 1 |
|  | SLC39A6 | | 1 |
|  | SLC44A4 | | 1 |
|  | TACSTD2 | | 1 |
| **Multiple Myeloma** |  | | **41** |
|  | TNFRSF17 | | 34 |
|  | CD38 | | 2 |
|  | ERBB2 | | 2 |
|  | CD19 | | 1 |
|  | CD70 | | 1 |
|  | TNFRSF8 | | 1 |
| **Ovarian** |  | | **39** |
|  | ERBB2 | | 14 |
|  | AXL | | 4 |
|  | F3 | | 4 |
|  | NECTIN4 | | 2 |
|  | CEACAM5 | | 1 |
|  | CLDN18 | | 1 |
|  | EPHA2 | | 1 |
|  | EGFR | | 1 |
|  | FGFR2 | | 1 |
|  | IL2RA | | 1 |
|  | KAAG1 | | 1 |
|  | LYPD3 | | 1 |
|  | MUC1 | | 1 |
|  | MUC16 | | 1 |
|  | ROR2 | | 1 |
|  | TNFRSF8 | | 1 |
|  | TPBG | | 1 |
|  | TACSTD2 | | 1 |
|  | VTCN1 | | 1 |
